# Artificial Intelligence Versus Rules-Based Approach for Segmenting NonPerfusion Area in a DRCR Retina Network Optical Coherence Tomography Angiography Dataset

**DOI:** 10.1167/iovs.66.3.22

**Published:** 2025-03-10

**Authors:** Tristan T. Hormel, Wesley T. Beaulieu, Jie Wang, Jennifer K. Sun, Yali Jia

**Affiliations:** 1 Oregon Health and Science University, Portland, Oregon, United States; 2 Jaeb Center for Health Research, Tampa, Florida, United States; 3 Joslin Diabetes Center, Beetham Eye Institute, Harvard Department of Ophthalmology, Boston, Massachusetts, United States

**Keywords:** artificial intelligence, image analysis, OCT angiography, diabetic retinopathy, nonperfusion area

## Abstract

**Purpose:**

Loss of retinal perfusion is associated with both onset and worsening of diabetic retinopathy (DR). Optical coherence tomography angiography is a noninvasive method for measuring the nonperfusion area (NPA) and has promise as a scalable screening tool. This study compares two optical coherence tomography angiography algorithms for quantifying NPA.

**Methods:**

Adults with (*N* = 101) and without (*N* = 274) DR were recruited from 20 U.S. sites. We collected 3 × 3-mm macular scans using an Optovue RTVue-XR. Rules-based (RB) and deep-learning–based artificial intelligence (AI) algorithms were used to segment the NPA into four anatomical slabs. For comparison, a subset of scans (*n* = 50) NPA was graded manually.

**Results:**

The AI method outperformed the RB method in intersection over union, recall, and F1 score, but the RB method has better precision relative to manual grading in all anatomical slabs (all *P* ≤ 0.001). The AI method had a stronger rank correlation with Early Treatment of Diabetic Retinopathy Study DR severity than the RB method in all slabs (all *P* < 0.001). NPAs graded using the AI method had a greater area under the receiver operating characteristic curve for diagnosing referable DR than the RB method in the superficial vascular complex, intermediate capillary plexus, and combined inner retina (all *P* ≤ 0.001), but not in the deep capillary plexus (*P* = 0.92).

**Conclusions:**

Our results indicate that output from the AI-based method agrees better with manual grading and can better distinguish between clinically relevant DR severity levels than a RB approach using most plexuses.

Diabetic retinopathy (DR) is the leading cause of blindness for working-age people in the United States.^1^ Vision loss associated with the disease can usually be prevented with timely intervention.^2^ However, DR is often asymptomatic, even as pathology develops.^3^ Accurate screening is therefore of paramount concern for DR management. Still, the gold standard for staging DR, the Early Treatment of Diabetic Retinopathy Study (ETDRS) DR Severity Scale is unwieldy for application in regular clinical practice.^4^

In the 30 years since the ETDRS DR Severity Scale was developed, optical coherence tomography angiography (OCTA) was introduced into clinical practice. This technique, based on motion contrast measurements between successive structural OCT B-scans, enables noninvasive vascular imaging at the capillary level and is capable of imaging separate vascular plexuses in isolation.^5^ Several retinal pathologies are quantifiable using OCTA,^6^^–^^8^ including the nonperfusion area (NPA), a clinically important feature in DR.^9^^–^^17^ In one cross-sectional study, higher NPA values were correlated with more severe DR.^13^ Importantly, evidence suggests that NPA assessment may be less dependent on good image quality (signal strength index [SSI]) than vessel density,^18^ currently the most commonly used OCTA metric for assessing perfusion.^19^

Trustworthy automated NPA measurements are essential for DR screening. The alternative, manual quantification is arduous and can be prohibitively time consuming. Automation, however, is complicated by the need for algorithms to identify NPA correctly in the presence of OCTA artifacts, which can alternately cause vascularized regions to appear avascular and avascular regions to appear perfused.^20^ To establish clinical usefulness in the face of such complications, NPA measurement procedures require validation on large, multiclinic datasets. To this end, we investigated the performance of two automated NPA measurement approaches on a dataset collated by the DRCR Retina Network across 20 clinical sites. The first automated NPA measurement approach is a traditional rules-based (RB) method that segments NPA using filtering, thresholding, and distance maps.^13^^,^^14^ We refer to this method as the RB approach in this work. The second is an artificial intelligence (AI)-based method that relies on convolutional neural networks.^16^ Both can extract NPA measurements from en face images of the superficial vascular complex (SVC), intermediate and deep capillary plexuses (ICP and DCP, respectively), and the inner retina (which combines the SVC, ICP, and DCP in a single en face image). We investigated the correlation and bias between the methods, the ability of each approach to classifying eyes into clinically relevant categories for DR management, and the robustness of each.

## Methods

### Imaging and Preprocessing

This study used 3 × 3-mm macular OCTA. Scans of eyes diagnosed with DR were collected by the DRCR Retina Network (protocols AA and W); scans of nondiabetic eyes were collected at Oregon Health and Science University (OHSU) through the studies supported by NEI R01 EY027833 and NIDDK DP3 DK104397. Eyes were grouped by DR severity into nonreferable (ETDRS score <35), referable (ETDRS score ≥35 to <53) and vision-threatening (ETDRS score ≥53) groups. All eyes were imaged by a 70-kHz commercial device (RTVue-XR, Optovue, Inc., Fremont, CA, USA) with 304 A-lines per B-scan, and 304 B-scans per volume. Flow signal was generated using split-spectrum amplitude-decorrelation angiography developed by Jia et al.^21^ Motion artifacts were corrected by merging x-fast and y-fast scans.^22^ Low-quality scans, defined as scans that included notable defocus or strong artifacts, were excluded from the analysis.^20^ A final criteria is the SSI, a manufacturer provided assessment of image quality. Although the AI-based method can provide accurate NPA measurements at lower signal strength, the RB method was not optimized for an SSI of <55, so for fairness we used this value as a cutoff for exclusion.

### Inclusion Criteria and Study Groups

Participants with DR were enrolled by DRCR Retina Network clinical sites for DRCR protocols AA and W. Eligibility criteria for study eyes included no substantial non-DR intraocular pathology (e.g., coincident AMD), no substantial media opacities, ETDRS DR Severity Scale score between 35 and 53 (inclusive), no history of panretinal photocoagulation or vitrectomy, no major ocular surgery within the prior 4 months, no treatment with intravitreal agents in the prior 12 months, and no center-involved diabetic macular edema. Nonstudy eyes from participants enrolled in the DRCR trials and eyes from participants who consented and were screened but did not enroll were also included. Both eyes from single individuals were included when eligible.

Healthy volunteers were enrolled at OHSU and underwent clinical examination to confirm the absence of diabetic retinopathy (ETDRS score = 10) or any other retinal pathology.

### RB NPA

The first approach examined in this report is a conventional algorithm using a set of rules to segment NPA (i.e., it is a RB method). Details of this RB algorithm have been published previously.^13^^,^^14^ Briefly, this approach uses a vesselness filter to enhance the vasculature in a plexus or complex.^23^ These vessel-enhanced images are then binarized with adaptive thresholding based on the reflectance signal. Next, a Euclidean distance transformation to the binarized image is used, which produces a vessel distance map in which each pixel value represents the distance to the nearest vessel. The NPA was finally determined by binarizing the distance map using a threshold value (determined by referencing healthy controls to ensure that no NPA was detected outside the fovea) and applying morphological operations to prevent small, nonpathological regions from being identified as NPA. Owing to the signal compensation steps to remove shadow artifact effects, the superiority of this method to the instrument-embedded method has been presented in previous publications.^13^^,^^14^^,^^18^

### Deep Learning-Based NPA

The deep learning model used in this work is a segmentation neural network based on a U-net architecture.^24^ A detailed description of the algorithm has been previously published.^15^^,^^25^^–^^28^ Model inputs consist of structural and angiographic en face images of the relevant slabs (the SVC, ICP, and DCP). The structural OCT input is used for differentiating the pixel with real nonperfusion feature (low OCTA value, but high OCT value) from the shadow artifact (low OCTA value and low OCT value). The algorithm determines the probability that a given pixel is NPA, and a segmented image is obtained by counting a pixel as NPA if the output confidence is >0.5.

Both the RB approach and the AI-based method were implemented in a custom designed software platform, Center for Ophthalmic Optics and Lasers Advanced Reading Toolkit (COOL-ART; version 2.0).^29^

### Algorithm Comparisons

To ensure unbiased results, each algorithm's performance was characterized using unseen data that were not used for algorithm development. Specifically, each algorithm was developed on data collected exclusively at OHSU, and evaluation was performed exclusively on DRCR data supplemented with new, unseen scans of healthy volunteers collected at OHSU. All comparisons were made on en face images projected across four different anatomical slabs frequently analyzed with OCTA: the SVC (defined as the inner 80% of the ganglion cell complex); ICP (defined as the outer 20% of the ganglion cell complex through the inner one-half of the inner nuclear layer), DCP (outer one-half of the inner nuclear layer through the outer plexiform layer), and the inner retina (the slab containing all of the SVC, ICP, and DCP). A projection-resolved OCTA algorithm was applied to clean projection artifacts.^30^

We performed comparisons of algorithm output to manual grading, to investigate their segmentation accuracy. For these comparisons, randomly selected scans from individuals with DR were manually graded by a masked grader (T.T.H.) using a previously described interface^16^ that allows for selection of NPA regions and exclusion of regions affected by artifacts such as shadowing. The results were used to calculate mean intersection over union, precision, recall, and F1 score with respect to algorithm outputs.

### Statistical Analyses

Differences in agreement metrics between the RB and AI methods and differences in NPA between manually graded and RB or AI methods were analyzed using a paired samples *t* test. Linear correlation coefficients and 95% confidence intervals (CIs) were estimated using a mixed effects linear model with random participant intercepts and an unstructured residual correlation matrix; CIs were estimated via bootstrapping (minimum of 10,000 replicates). Receiver operating characteristic curves, differences, and 95% CIs were estimated using logistic regression with cluster bootstrapping (10,000 replicates). These analyses were limited to one scan per eye; for eyes that had multiple scans, the first scan was used for analysis.

Differences in the mean SSI and NPA by DR severity were estimated from linear mixed models with random intercepts for participants to account for participants with both eyes included and an unstructured residual covariance matrix to account for eyes scanned at multiple visits. Up to three scans per eye were included when available.

There was no control of the type 1 error rate in these exploratory analyses, and some associations may have been identified owing to chance. With this caveat, *P* values of ≤0.05 were considered statistically significant. All analyses were conducted using SAS software, Version 9.4 (SAS Institute Inc., Cary, NC, USA).

## Results

### Study Population

**Figure 1.**
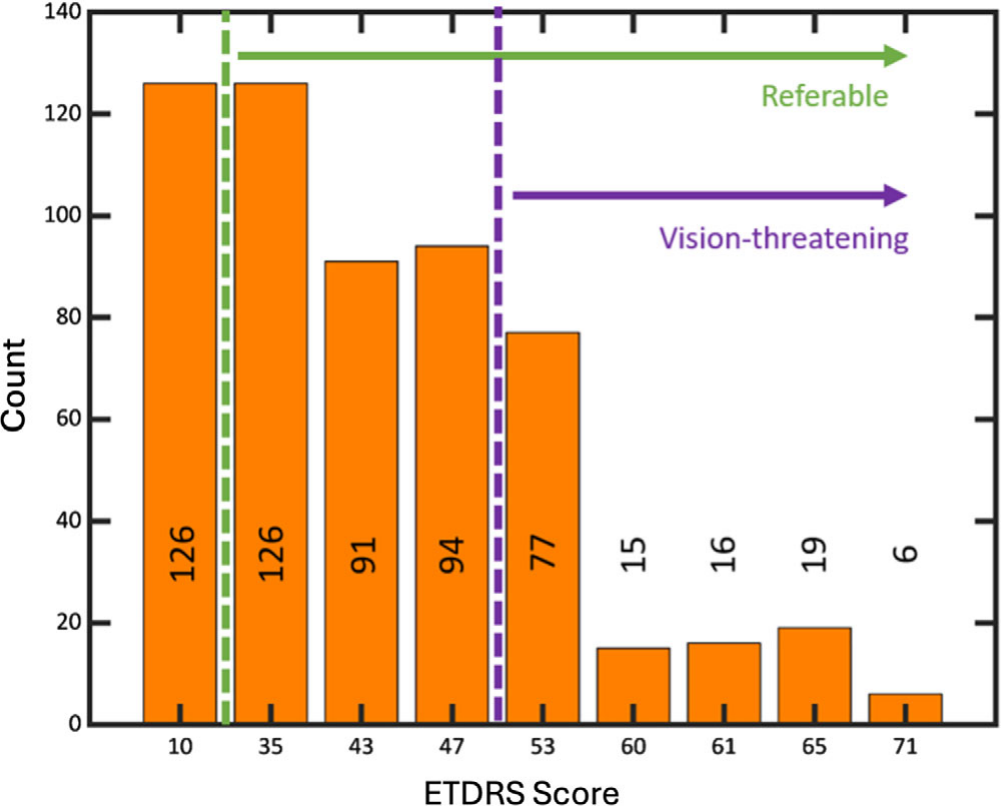
DR severity in eyes at first visit. DR severity in eyes at the first visit (570 eyes from 375 participants). Dashed lines indicate cutoff values for referable (ETDRS level ≥ 35) and vision-threatening DR (ETDRS level ≥53). Most scans in this study were of eyes with ETDRS scores between 35 and 47, which belong to referable but non–vision-threatening DR. An ETDRS score of 10 corresponds to data from volunteers from OSHU who do not have diabetes (ETDRS level = 10, no retinopathy). Numbers in or above the bars indicate the number of eyes at each score.

The main study population consisted of 375 participants (570 eyes) with 819 visits (1 scan per visit). Among the participants, 191 (51%) were male and 180 (48%) were female (4 [1%] not reported), with an average age of 57 ± 16 years. The race/ethnicity of the participants was as follows: 258 (69%) White, 57 (15%) Black or African American, 26 (7%) Hispanic or Latino, 24 (6%) Asian, 1 (<1%) American Indian/Alaska, 1 (<1%) more than one race, and 8 (2%) unknown or not reported. At the first available visit, 126 eyes (22%) had nonreferable DR (below level 35), 311 (55%) had referable but non–vision-threatening DR (level 35 to <53), and 133 (23%) had vision-threatening DR (level 53 or higher) (Fig. 1).

### Agreement of RB and AI Methods With Manual Segmentation

We measured the intersection over union, precision, recall, and F1 score for each method's output compared to a manually segmented “ground truth.” We note that, without histopathological correlation, we cannot say a priori that the manually segmented result reflects the underlying anatomy better or worse than either of the algorithm outputs. The comparisons in this section should be interpreted as indicating how similar algorithm outputs are to expert human grading. We found that, in each layer, the AI-based method outperformed the RB approach in intersection over union, recall, and F1 score, but the RB method achieved better precision (all *P* ≤ 0.001) (Table 1). Relative to the manually graded images, differences in intersection over union, recall, and F1 score all increased to the benefit of the AI approach when more NPA was recognized by the manual grader, while differences in precision were relaxed (Supplementary Table S1).

**Table 1.** Algorithm Agreement With Manual Segmentation by Method

		AI Method		RB Method			
Metric	Slab	No.^a^	Mean ± SD	No.^a^	Mean ± SD	Difference (95% CI)	*P* Value
IOU	SVC	49	0.66 ± 0.08	49	0.44 ± 0.10	0.21 (0.18 to 0.24)	<0.001
	ICP	49	0.69 ± 0.15	49	0.43 ± 0.21	0.26 (0.21 to 0.31)	<0.001
	DCP	49	0.76 ± 0.12	49	0.42 ± 0.22	0.34 (0.28 to 0.40)	<0.001
	Inner Retina	49	0.72 ± 0.11	49	0.48 ± 0.19	0.24 (0.18 to 0.30)	<0.001
Precision	SVC	49	0.77 ± 0.11	49	0.81 ± 0.09	−0.04 (−0.06 to −0.01)	0.001
	ICP	49	0.84 ± 0.13	48	0.97 ± 0.04	−0.13 (−0.17 to −0.09)	<0.001
	DCP	49	0.90 ± 0.13	49	0.97 ± 0.07	−0.07 (−0.11 to −0.04)	<0.001
	Inner Retina	49	0.78 ± 0.12	48	1.00 ± 0.01	−0.22 (−0.25 to −0.18)	<0.001
Recall	SVC	49	0.83 ± 0.08	49	0.51 ± 0.13	0.32 (0.29 to 0.35)	<0.001
	ICP	49	0.81 ± 0.15	49	0.44 ± 0.21	0.37 (0.32 to 0.43)	<0.001
	DCP	49	0.86 ± 0.13	49	0.43 ± 0.23	0.43 (0.38 to 0.48)	<0.001
	Inner Retina	49	0.91 ± 0.06	49	0.48 ± 0.19	0.43 (0.38 to 0.48)	<0.001
F1 score	SVC	49	0.79 ± 0.06	49	0.61 ± 0.09	0.18 (0.15 to 0.21)	<0.001
	ICP	49	0.81 ± 0.12	48	0.59 ± 0.19	0.22 (0.17 to 0.27)	<0.001
	DCP	49	0.86 ± 0.09	49	0.56 ± 0.22	0.30 (0.24 to 0.36)	<0.001
	Inner Retina	49	0.83 ± 0.08	48	0.64 ± 0.17	0.19 (0.14 to 0.25)	<0.001

IOU, mean intersection over union.

a Sample size is 50 eyes from 50 individuals. Precision was undefined for the ruled-based method in the ICP and inner slabs because both the manually graded and RB methods did not identify any nonperfusion.

**Figure 2.**
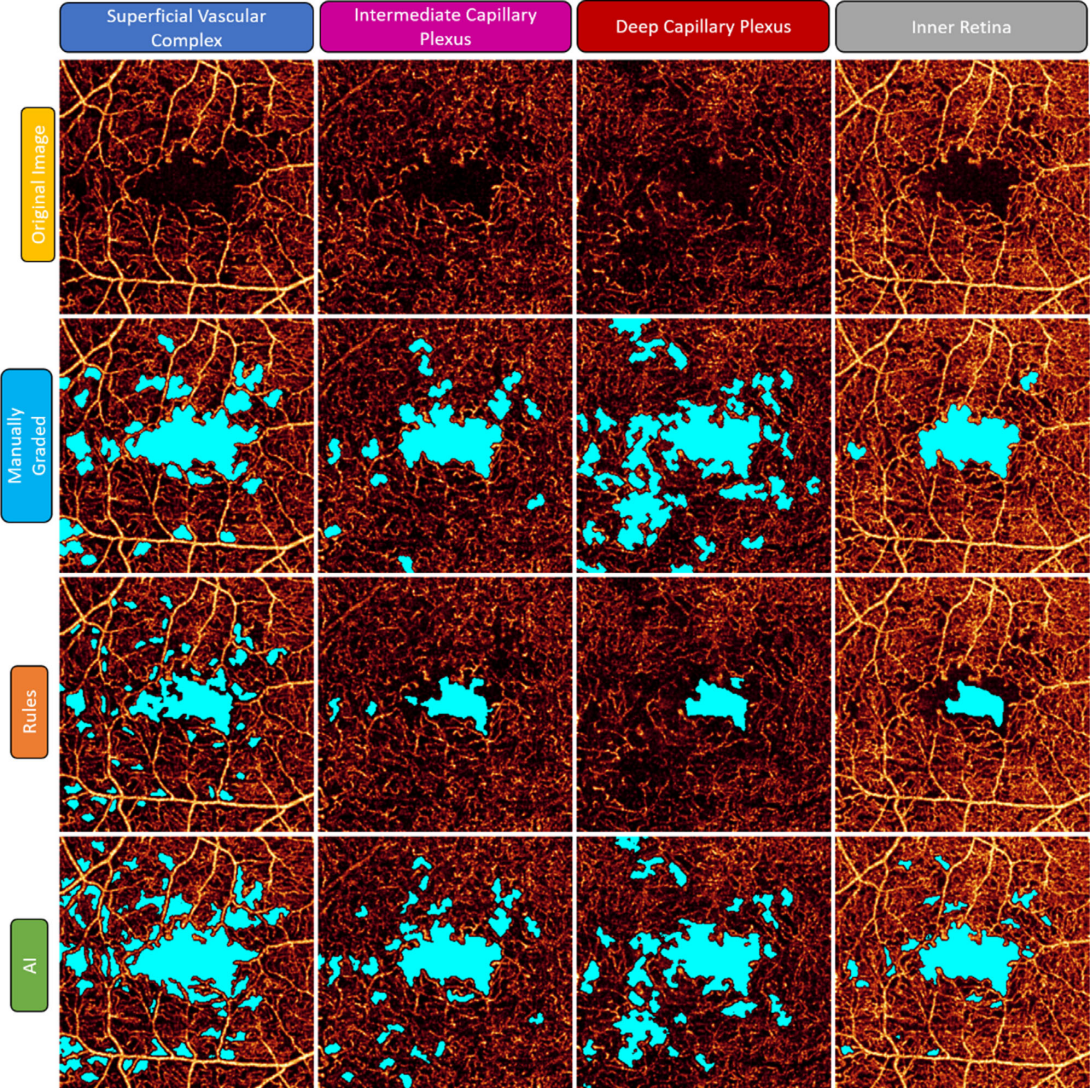
Manual segmentation and algorithm outputs. Relative to manual segmentation, the RB algorithm fails to identify some regions of the NPA, possibly owing to the relatively high background noise (see especially the DCP and inner retina images). The AI result, on the other hand, broadly agrees with the general location of NPA regions; however, the specific shape of these regions is not necessarily consistent. The AI result also identifies small NPA regions that were not manually segmented truth image (again, compare especially the inner retina images).

These results are consistent with a visual comparison of the outputs (Fig. 2). Differences in the AI output from manual segmentation appear to mainly reflect (1) the borders of large NPA regions, although their location is broadly consistent, and (2) a propensity of the AI method to segment smaller NPA regions than were identified by the grader. In contrast, the RB method failed to identify some NPA in regions with a strong background.

In the SVC, both the RB and AI methods had good agreement with manual grading with an intraclass correlation coefficient of 0.88 (95% CI, 0.64–0.95) for RB and 0.85 (95% CI, 0.30–0.95) for AI (Supplementary Fig. S1, Supplementary Table S2). In the ICP, DCP, and combined inner retina slabs, the RB agreement with manual grading decreased whereas the AI method maintained good agreement, on average: 0.67 (95% CI, 0.13–0.86) vs. 0.86 (95% CI, 0.76–0.92) in the ICP, 0.58 (95% CI, 0.21–0.78) vs. 0.92 (95% CI, 0.86–0.95) in the DCP, and 0.54 (95% CI, 0.02–0.78) vs. 0.87 (95% CI, 0.15–0.96) in the combined inner retina.

In every slab, the RB method identified less NPA than the manual method with differences of −0.23 mm^2^ (95% CI, −0.32 to −0.14 mm^2^), −0.21 mm^2^ (95% CI, −0.28 to −0.15 mm^2^), −0.25 mm^2^ (95% CI, −0.35 to −0.14), and −0.20 mm^2^ (95% CI, −0.26 to −0.14 mm^2^) in the SVC, ICP, DCP, and combined inner retina, respectively (all *P* < 0.001) (Supplementary Fig. S2). Conversely, the AI method tended to identify more NPA than the manual method with AI minus manual differences of 0.35 mm^2^ (95% CI, 0.25 to 0.44 mm^2^; *P* < 0.001), 0.06 mm^2^ (95% CI, −0.01 to 0.13 mm^2^; *P* = 0.07), −0.01 mm^2^ (95% CI, −0.07 to 0.05 mm^2^; *P* = 0.74), and 0.15 mm^2^ (95% CI, 0.12 to 0.19 mm^2^; *P* < 0.001) in the SVC, ICP, DCP, and inner retina slabs, respectively. For the RB method, the difference seemed to be larger in scans with more NPA detected by manual grading whereas the AI differences were more consistent across scans with varying amounts of NPA detected by manual grading (Supplementary Fig. S3).

### Agreement Between RB and AI Methods

**Figure 3.**
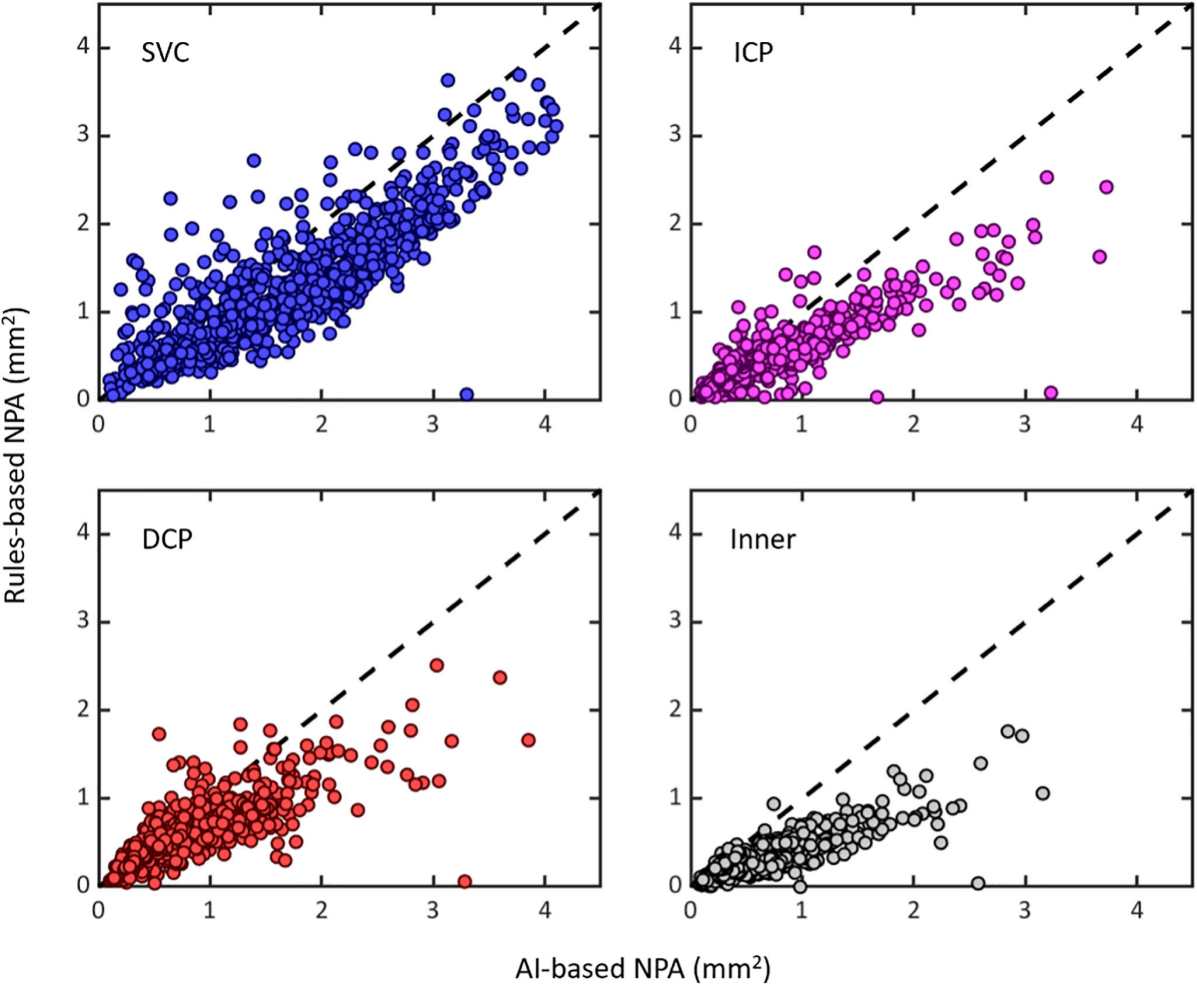
Correlation between AI and RB NPA measurements. The correlation coefficient measures the linear fit of the data. Sample size was 570 eyes (1 scan per eye) of 375 participants. All correlations were significant at an α level of 0.05. The dashed line represents the line of identity. The inner retina spans each of the other layers considered in this study.

**Figure 4.**
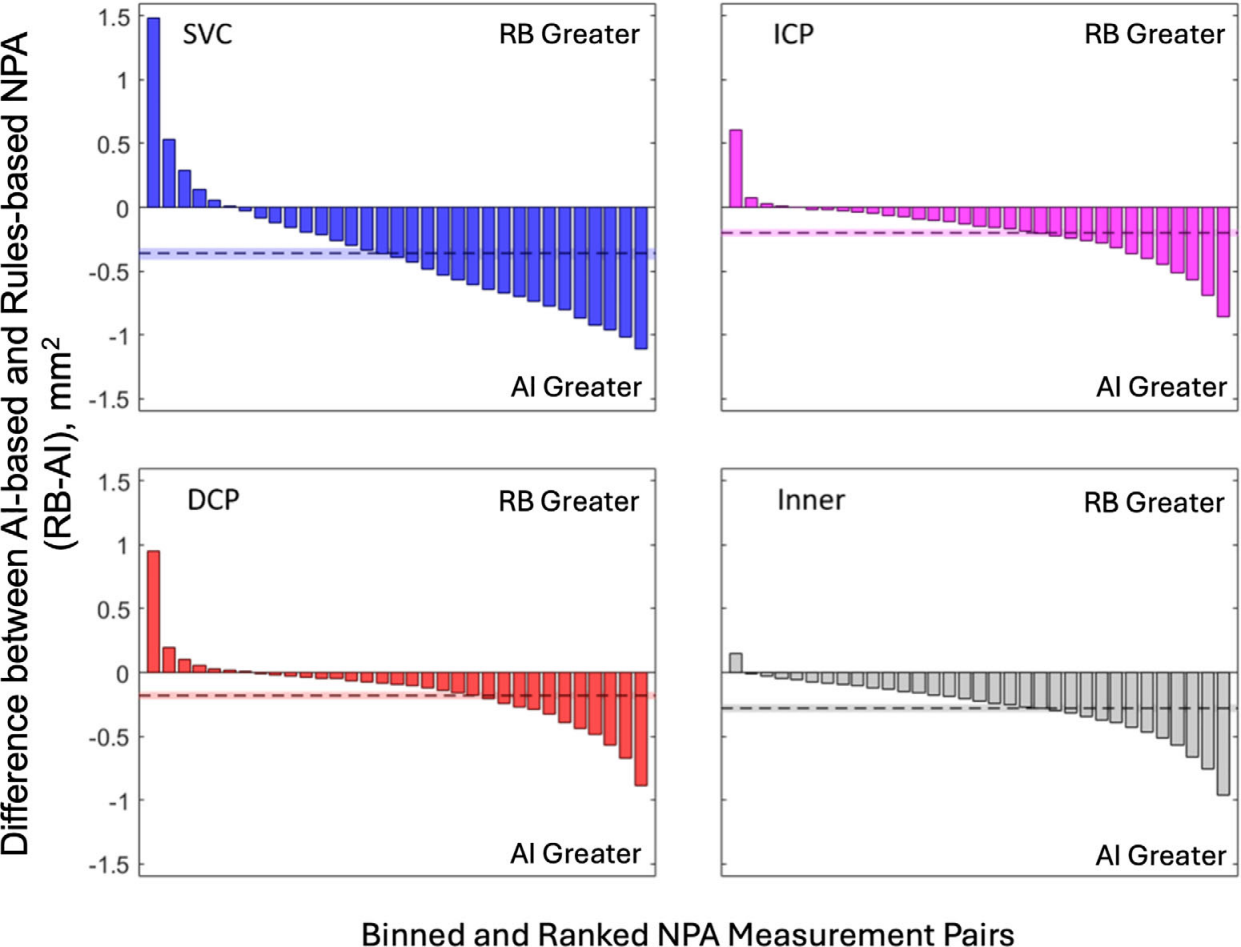
Difference in NPA between AI and RB methods by anatomical slab. Waterfall plots (showing binned averages of 25 measurements for clarity) showing the mean difference (dotted line, with CI given by the shaded region) between AI and RB NPA measurements (calculated as NPA with RB minus NPA from AI with negative values indicating greater NPA with the AI method). Sample size was 819 scans from 570 eyes of 375 participants for all anatomical slabs. All comparisons were significant at an α level of 0.05.

NPA measurements made with both methods were highly correlated, with linear correlation coefficients of 0.86 (95% CI, 0.82–0.89), 0.84 (95% CI, 0.74–0.91), 0.79 (95% CI, 0.70–0.86), and 0.81 (95% CI, 0.71–0.87) in the SVC, ICP, DCP, and combined inner retina, respectively (Fig. 3). The RB approach on average, however, found less NPA across all three vascular layers than the AI method with RB minus AI mean differences of −0.36 (95% CI, −0.41 to −0.32), −0.20 (95% CI, −0.23 to −0.17), −0.18 (95% CI, −0.21 to −0.15), and −0.28 (95% CI, −0.31 to −0.25) mm^2^ in the SVC, ICP, DCP, and combed inner retina, respectively (Fig. 4) (all *P* < 0.001). This difference in means resulted in lower agreement between the methods with intraclass correlation coefficients of 0.76 (95% CI, 0.47–0.87), 0.66 (95% CI, 0.38–0.80), 0.65 (95% CI, 0.45–0.77), 0.46 (95% CI, 0.03–0.70) in the SVC, ICP, DCP, and combined inner retina, respectively.

### Reliability of RB and AI Methods

**Figure 5.**
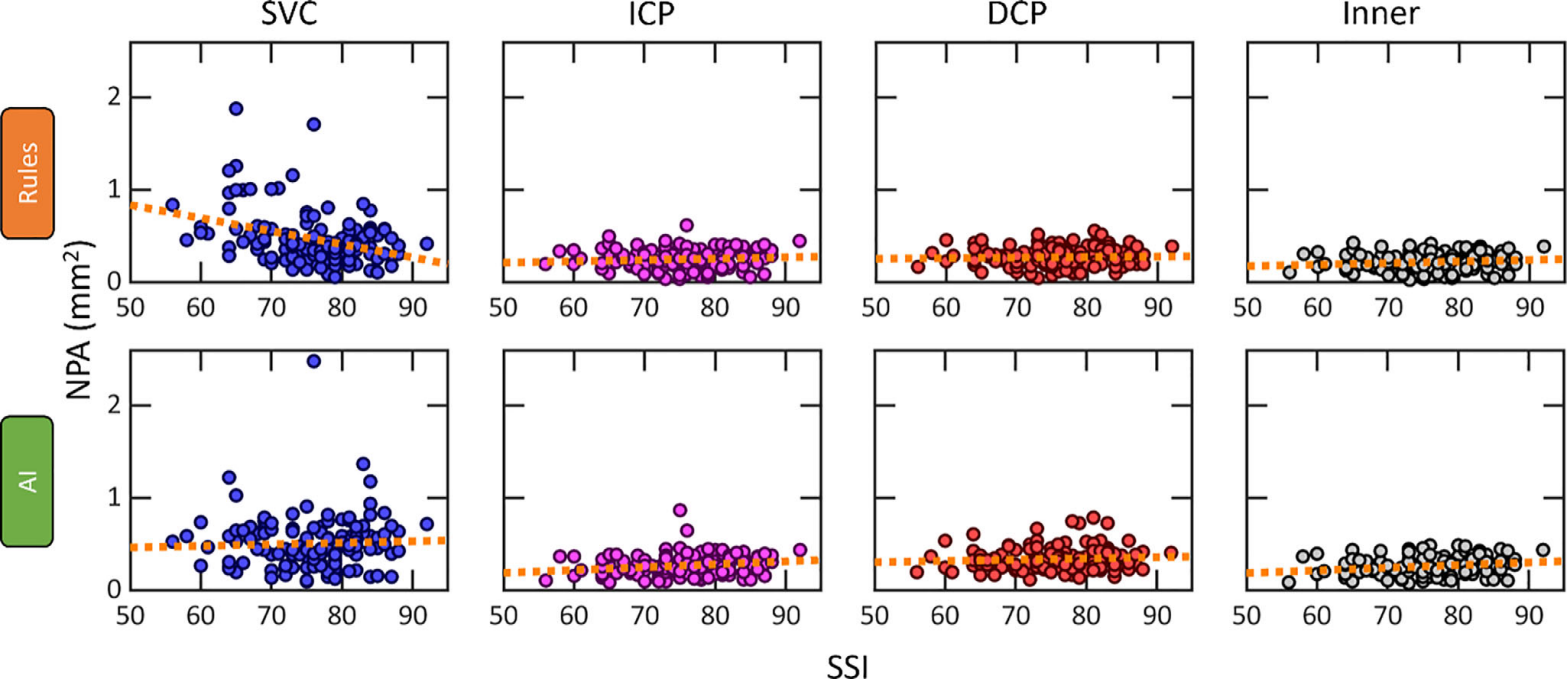
Correlation between NPA and image quality by method. Correlation between NPA measurements and image quality (SSI) among eyes from participants without diabetes (125 eyes from 101 participants). Ideally, the SSI and NPA would be uncorrelated in this context.

To determine the effects of signal quality on measurements, we correlated NPA measurements with SSI. Although the SSI provides a means to quantify image quality, it does not directly indicate the presence of some features including motion, signal reduction artifacts, or image defocus. Images from patients with more advanced stages of DR are more likely to include tissue thinning and atrophy, which may also affect the SSI, in which case SSI variation would exist even with ideal imaging conditions. This result means that some variation in NPA values with respect to SSI may reflect real physiological differences. In this dataset, the mean SSI was significantly greater in scans from eyes with nonreferable DR compared with referable DR (difference = 8.4; 95% CI, 6.9–9.9; *P* < 0.001) and vision-threatening DR (difference = 8.8; 95% CI, 7.2–10.5; *P* < 0.001). Therefore, we avoided the confounding influence of DR severity by evaluating the NPA vs. SSI correlations among eyes from participants without diabetes (Fig. 5; Table 2).

**Table 2.** Linear Correlations Between NPA and Image Quality by DR Severity in Eyes Without Diabetes

	Method^a^ (*N* = 125 Eyes, 101 Participants)		
Anatomical Slab	AI	RB	Difference (95% CI)
SVC	0.09 (−0.06, 0.25)	**−0.31 (−0.45 to −0.16)**	**0.41 (0.24 to 0.60)**
ICP	**0.20 (0.04, 0.37)**	0.09 (−0.09 to 0.27)	**0.12 (0.02 to 0.20)**
DCP	0.09 (−0.07, 0.24)	0.04 (−0.14 to 0.21)	0.06 (−0.07 to 0.18)
Inner retina	**0.20 (0.02, 0.38)**	0.11 (−0.07 to 0.29)	**0.09 (0.03 to 0.16)**

a Values in parentheses are 95% CIs. Bolded correlations in grayed cells are significant at an α level of 0.05. Correlations were estimated from a linear mixed model to account for nonindependence of eyes from the same participant.

In the SVC, NPA measured by the RB method had significant negative correlation with SSI (r = −0.31; 95% CI, −0.45 to −0.16) whereas NPA measured by the AI method was not significantly correlated with the SSI (r = 0.09; 95% CI, −0.06 to 0.25) (Fig. 5; Table 2). A negative correlation indicates that scans with greater SSI (better image quality) were graded as having less NPA, on average. In the ICP and inner retina, there were significant positive correlations between SSI and NPA for the AI method (0.20 [95% CI, 0.04 to 0.37], and 0.20 [95% CI, 0.02 to 0.38], respectively), but not the RB method (0.09 [95% CI, −0.09 to 0.27] and 0.11 [95% CI, −0.07 to 0.29], respectively). A significant positive correlation indicates that eyes with a greater SSI were graded as having more NPA, on average. Neither method exhibited a significant correlation between the SSI and NPA in the DCP.

### Diagnosis and Staging of DR

**Figure 6.**
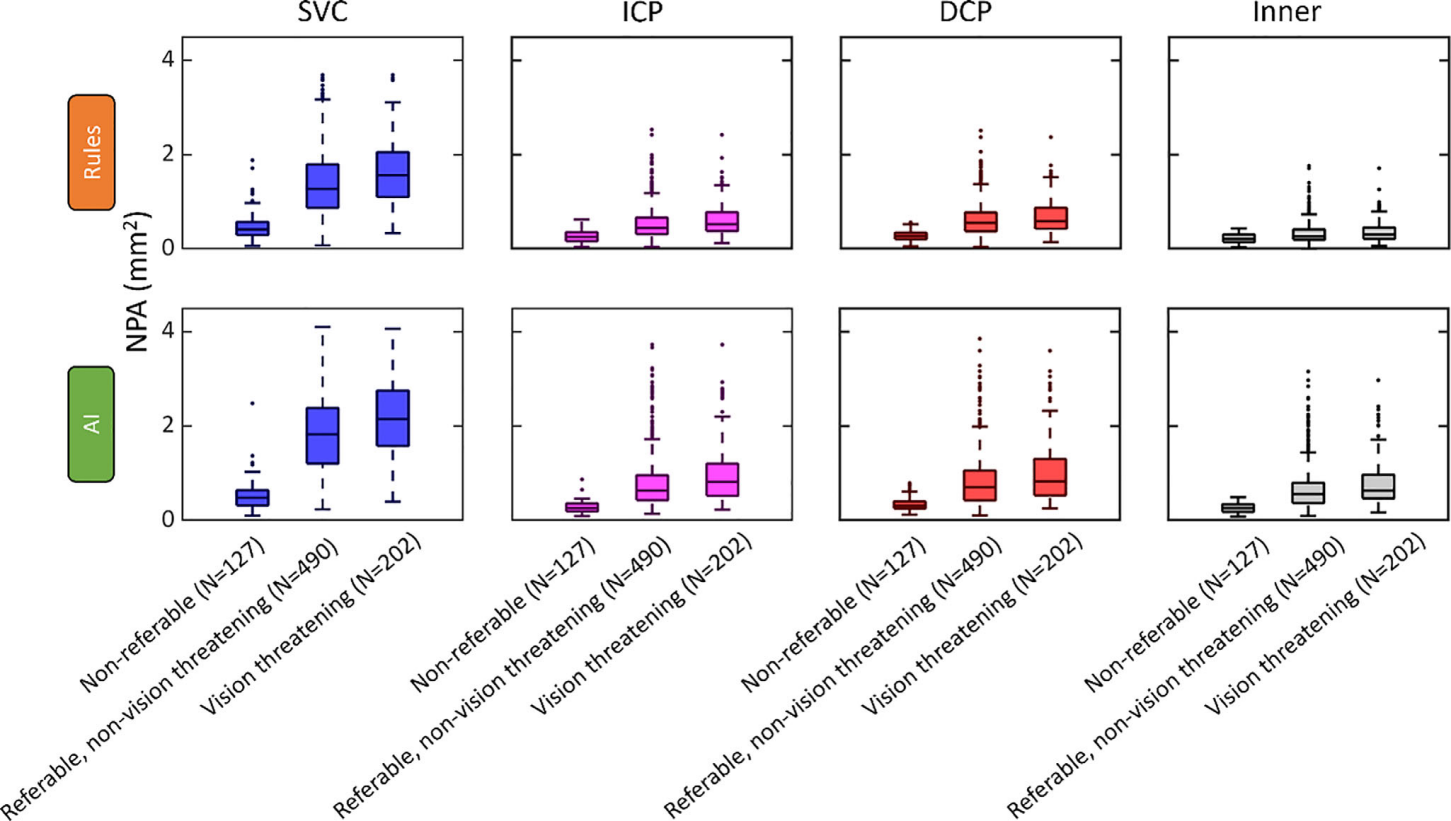
NPA by DR severity. Box plots of the NPA by DR severity stratified by method (*rows*) and anatomical slab (slab). Both methods show an overlap between the NPA and disease severity. The inner retina includes each of the slabs considered in this study. The sample size was 127 scans of 127 eyes from 103 participants for nonreferable DR (ETDRS level <35), 490 scans of 361 eyes from 239 participants for referable but non–vision-threatening DR (ETDRS levels 35 to <53), and 202 scans of 171 eyes from 126 participants for vision-threatening DR (ETDRS level ≥53).

We assessed the usefulness of the RB and AI NPA measurement methods for clinical practice by associating each measurement with DR severity. Categories included nonreferable (ETDRS level <35), referable but non–vision-threatening DR (ETDRS level ≥35 to <53), and vision-threatening DR (ETDRS level ≥53). We found an appreciable overlap in NPA measurements between these categories. Both the RB and AI methods detected less NPA in eyes with nonreferable DR compared to eyes with referable and/or vision-threatening DR (Fig. 6; Table 3). Differences in NPA between eyes with vision-threatening DR (≥ level 53) and referable but non–vision-threatening DR (levels 35 to <53) were smaller and not always statistically significant.

**Table 3.** NPA by DR Severity

			AI Method					RB Method				
Slab	DR Severity	No. of Scans^a^	Mean (95% CI)	Difference vs. NonReferable (95% CI)	*P* Value	Difference vs. Referable (95% CI)	*P* Value	Mean (95% CI)	Difference vs. NonReferable (95% CI)	*P* Value	Difference vs. Referable (95% CI)	*P* Value
SVC	Nonreferable	127	0.60 (0.47 to 0.73)					0.50 (0.39 to 0.61)				
	Referable	490	1.82 (1.74 to 1.90)	1.22 (1.07 to 1.38)	<0.001			1.37 (1.30 to 1.43)	0.87 (0.74 to 1.00)	<0.001		
	Vision-threatening	202	1.89 (1.79 to 1.98)	1.29 (1.13 to 1.45)	<0.001	0.07 (0.00 to 0.13)	0.05	1.49 (1.40 to 1.57)	0.99 (0.85 to 1.13)	<0.001	0.12 (0.05 to 0.19)	<0.001
ICP	Nonreferable	127	0.32 (0.23 to 0.40)					0.27 (0.21 to 0.32)				
	Referable	490	0.78 (0.73 to 0.84)	0.47 (0.36 to 0.57)	<0.001			0.52 (0.48 to 0.55)	0.25 (0.18 to 0.31)	<0.001		
	Vision-threatening	202	0.81 (0.75 to 0.86)	0.49 (0.39 to 0.59)	<0.001	0.02 (−0.01 to 0.05)	0.11	0.54 (0.50 to 0.58)	0.27 (0.20 to 0.34)	<0.001	0.02 (−0.01 to 0.06)	0.19
DCP	Nonreferable	127	0.37 (0.28 to 0.46)					0.28 (0.23 to 0.34)				
	Referable	490	0.82 (0.76 to 0.87)	0.45 (0.35 to 0.55)	<0.001			0.62 (0.58 to 0.65)	0.33 (0.27 to 0.40)	<0.001		
	Vision-threatening	202	0.90 (0.84 to 0.96)	0.53 (0.42 to 0.64)	<0.001	0.08 (0.04 to 0.12)	<0.001	0.65 (0.61 to 0.69)	0.37 (0.30 to 0.44)	<0.001	0.04 (−0.00 to 0.07)	0.052
Inner	Nonreferable	127	0.31 (0.24 to 0.38)					0.23 (0.20 to 0.27)				
	Referable	490	0.66 (0.62 to 0.71)	0.35 (0.27 to 0.43)	<0.001			0.31 (0.29 to 0.34)	0.08 (0.04 to 0.12)	<0.001		
	Vision-threatening	202	0.67 (0.62 to 0.71)	0.35 (0.27 to 0.44)	<0.001	0.00 (−0.02 to 0.03)	0.76	0.32 (0.30 to 0.35)	0.09 (0.05 to 0.14)	<0.001	0.01 (−0.01 to 0.03)	0.30

a Scans included 127 eyes from 103 participants for nonreferable, 361 eyes from 239 participants for referable, and 171 eyes from 126 participants for vision-threatening.

To measure the association between DR severity and NPA, we used the nonparametric Spearman rank correlation coefficient because the ETDRS DR severity scale is ordinal (discrete levels and the absolute spacing between levels is noninformative). For both methods, the correlation was highest in the SVC, decreased through the ICP and DCP, and was lowest in the combined inner retina (Supplementary Fig. S4; Table 4). The AI method had a stronger correlation with DR severity than the RB method in each slab: 0.65 vs. 0.59 in the SVC (difference = 0.06; 95% CI, 0.02–0.10), 0.58 vs. 0.45 in the ICP (difference = 0.13; 95% CI, 0.10–0.16), 0.54 to 0.49 in the DCP (difference = 0.05; 95% CI, 0.02–0.09), and 0.53 vs. 0.23 in the combined inner retina (difference = 0.31; 95% CI, 0.26–0.36).

**Table 4.** Rank Correlations Between NPA DR Severity

	Method^a^ (*N* = 570 Eyes, 375 Participants)		
Anatomical Slab	AI	RB	Difference (95% CI)
SVC	0.65 (0.60–0.69)	0.59 (0.53–0.64)	0.06 (0.02–0.10)
ICP	0.58 (0.53–0.63)	0.45 (0.39–0.51)	0.13 (0.10–0.16)
DCP	0.54 (0.48–0.60)	0.49 (0.42–0.55)	0.05 (0.02–0.09)
Inner retina	0.53 (0.47–0.59)	0.23 (0.15–0.30)	0.31 (0.26–0.36)

a Values in parentheses are 95% CIs. All correlations and differences in correlations between methods were significant at an α level of 0.05. Correlations were estimated from a linear mixed model to account for nonindependence of eyes from the same participant.

**Figure 7.**
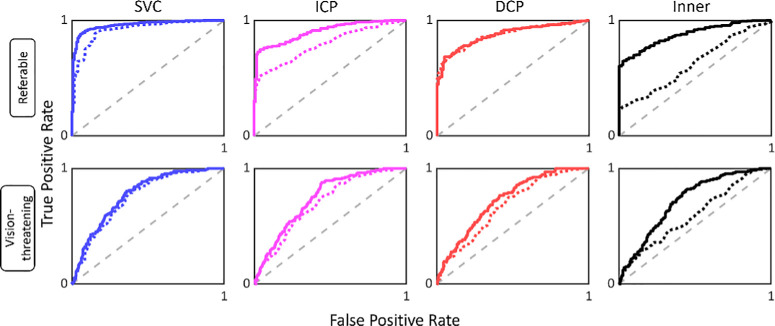
Received operator characteristic curves for referable and vision-threatening DR. Receiver operating characteristic curves for referable and vision-threatening DR diagnosis. The sample size is 570 eyes from 375 participants. The AI method (*solid lines*) is in general better than the RB approach (*dotted lines*) at diagnosing these DR severities, in particular when the inner retina images are used for staging.

These measurements indicate that NPA measurements reflect DR severity, but do not speak to the measurements’ ability to stage clinically important thresholds of DR severity. To investigate, we attempted to stage DR according to two clinically relevant categories: nonreferable vs. referable (ETDRS level ≥35, which includes vision-threatening DR), and non–vision-threatening DR (including referable) vs. vision threatening (ETDRS level ≥53). We found that the AI-based method broadly outperformed the RB method for diagnostics, and especially in whole inner retina images (Fig. 7; Table 5). Specifically, the area under the receiver operating characteristic curve (AUROC) for diagnosing referable DR in the SVC was 0.959 (95% CI, 0.937–0.975) for the AI method and 0.919 (95% CI, 0.888–0.946) in the RB method, which is a difference of 0.039 (95% CI, 0.016–0.065; *P* = 0.001). Differences in AUROC for referable DR were also greater with AI than RB in the ICP and inner retina (both *P* < 0.001) but not the DCP (*P* = 0.92). For diagnosing vision-threatening DR, the AUROC in the SVC was 0.772 (95% CI, 0.728 to 0.814) with AI and 0.751 (95% CI, 0.705 to 0.794) with RB for a difference of 0.021 (95% CI, −0.005 to 0.046, *P* = 0.11). Differences in AUROC for vision-threatening DR were greater with AI than RB In the ICP, DCP, and inner retina (all *P* ≤ 0.001).

**Table 5.** Staging DR by NPA Measurements

		Area Under the Receiver Operator Characteristic Curve (95% CI)			
Outcome	Anatomical Slab^a^	AI	RB	Difference (95% CI)	*P* Value (Delong's Method)
Referable DR (≥level 35)	SVC	0.959	0.919	0.039	0.001
		(0.937 to 0.975)	(0.888 to 0.946)	(0.016 to 0.065)	
	ICP	0.904	0.792	0.112	<0.001
		(0.871 to 0.931)	(0.745 to 0.836)	(0.079 to 0.143)	
	DCP	0.876	0.875	0.001	0.92
		(0.839 to 0.909)	(0.840 to 0.906)	(−0.023 to 0.025)	
	Inner retina	0.879	0.628	0.250	<0.001
		(0.843 to 0.910)	(0.562 to 0.690)	(0.211 to 0.292)	
Vision-threatening DR (≥level 53)	SVC	0.772	0.751	0.021	0.11
		(0.728 to 0.814)	(0.705 to 0.794)	(−0.005 to 0.046)	
	ICP	0.743	0.699	0.044	<0.001
		(0.696 to 0.787)	(0.648 to 0.748)	(0.018 to 0.070)	
	DCP	0.724	0.670	0.053	<0.001
		(0.672 to 0.772)	(0.616 to 0.724)	(0.026 to 0.081)	
	Inner retina	0.719	0.597	0.122	<0.001
		(0.667 to 0.767)	(0.536 to 0.656)	(0.085 to 0.161)	

a Sample size was 570 scans from 570 eyes (one scan per eye) of 375 participants for all anatomical slabs.

## Discussion

In this work, we compared the performance of two NPA measurement methods, an AI-based approach and an RB approach, in multiple anatomical slabs within the macula in nondiabetic eyes and eyes across the full DR severity spectrum.

Results from each method were highly correlated (Fig. 3). However, there were important differences in performance. Across each anatomical layer considered in this study, the AI method outperformed the RB method for mean intersection over union, recall, and F1 score relative to a manually graded ground truth, whereas the RB method outperformed the AI method in precision. This may be a consequence of the AI method's propensity to detect larger amounts of NPA relative to the RB method (Fig. 4). The improved precision is in part a consequence of the RB approach detecting a smaller amount of NPA; although human grading typically had high agreement with the NPA detected using this approach, it also failed to detect NPA revealed by the AI-based method that was labeled by a human grader. At least in some cases, this result seems to have been due to the RB method inadequately accounting for noise. Overall, the AI method measured larger NPA values than the RB approach, especially when both methods agreed that extensive NPA was present (Fig. 4). It is also worth noting that the difference in precision was small (−0.04 in the SVC, for example) relative to other metrics (0.21, 0.32, and 0.18 for intersection over union, recall, and F1 score, respectively, in the same slab).

Receiver operating characteristic curve analysis demonstrated better performance for the AI method in staging DR into referable and vision-threatening categories in most anatomical slabs considered (Fig. 7; Table 5). It should be noted, however, that neither of the NPA measurement methods in this study produced measurements that were capable of reliably staging vision-threatening DR vs. non–vision-threatening DR. This lack of reliable staging capability is likely a consequence of (1) the large variation in macular NPA metrics across eyes at a single clinical severity level (Fig. 6) and (2) the fact that high ETDRS scores (>53) reflect the presence of proliferation or other advanced pathology rather than larger amounts of NPA.^31^

Only the RB method's results are correlated with SSI in the SVC, the least artifact-prone slab in OCTA images and also among the most frequently analyzed (Fig. 5; Table 3). We have previously noted correlation with the RB approach on device output for low-quality (SSI of <55) scans,^13^^,^^18^^,^^32^ but here we also found this dependency in the output for the SVC. Conversely, the AI approach exhibited correlation with SSI in the ICP and DCP, although in this case the correlation was positive rather than negative. The negative correlation with the RB result and SSI in the SVC is easily explained as the RB failing to correctly interpret shadow areas and signal attenuation, issues that the AI method was trained specifically to account for. The positive correlation between the AI approach and SSI, and lack of negative correlation for the RB method for the reasons just given, are harder to interpret. However, the first thing to note is that the correlation in the AI method here is relatively smaller, and so evidences a smaller effect. We speculate that it is largely to do with better image quality granting the AI method more confidence in predictions along the edge of the foveal avascular zone. The RB method would not exhibit the same effect because it only detects regions with relatively high confidence, and the lack of a negative correlation could be explained by signal attenuation being present in the deeper layers, independent of image quality.

Both methods had performance differences within the different anatomical slabs examined in this study. The combined inner retina images were notably poorer than other slabs for staging referable and vision-threatening DR, especially with NPA measurements made using the RB approach (Fig. 7). Alternatively, staging using NPA within just the SVC produced the best results, perhaps owing to the relative clarity of the images. This could also reflect pathoanatomic patterns of capillary dropout. Judging by correlation with SSI in nonreferable eyes, which should not display as many confounding effects of disease, the RB method was also much more affected by noise in this slab. The ability of the AI-based approach to remain uncorrelated with SSI in the SVC while also achieving a large AUROC for referable DR diagnosis supports the notion that the AI-based method is more useful for DR analysis within this slab.

This study has limitations. We lacked both a large number of manually labeled images and repeat scans from which to assess measurement accuracy and reliability. Histopathological results to establish a ground truth from which to assess each algorithm's performance more objectively with respect to anatomical NPA distributions are also lacking, although it is difficult to see how these results could be obtained in a study similar to ours. We also note that both methods’ accuracy was lower relative to previously published results in which metrics were evaluated on datasets consisting of high-quality images.^13^^,^^14^^,^^16^ Although this factor may seem like a limitation, the previous results were developed and evaluated on the same dataset, which were both smaller and exhibited higher scan quality overall. This evaluation, in contrast, was on an unseen external dataset, and still shows the higher potential of AI method. In the future, the AI method should be given more attention for being adopted in clinical use.

## Conclusions

In general, AI-based NPA detection achieves better agreement with manual grading, is more resilient to image quality variation, and is more capable of staging DR into clinically relevant severities than a RB approach. Future studies may determine the ability of AI-determined NPA metrics to predict DR worsening or improvement over long-term follow-up.

## Supplementary Material

Supplement 1
