# Supplementary material for: Artificial Intelligence Versus Rules-Based Approach for Segmenting NonPerfusion Area in a DRCR Retina Network Optical Coherence Tomography Angiography Dataset
Source: Invest Ophthalmol Vis Sci. 2025 Mar 10;66(3):22. doi: 10.1167/iovs.66.3.22 (PMC11905605; doi:10.1167/iovs.66.3.22)
Supplement: Supplement 1 [file iovs-66-3-22_s001.pdf]

Online Table 1. Algorithm Agreement with Manual Segmentation by Method and Non-Perfusion Area.

Metric	Slab	Manually Graded NPA ≤ Median						Manually Graded NPA > Median						Interaction P
		AI		RB		Difference (95% CI)	P	AI		RB		Difference (95% CI)	P	
		N	Mean (SD)	N	Mean (SD)			N	Mean (SD)	N	Mean (SD)	N		
IOU	SVC	25	0.62 (0.08)	25	0.46 (0.10)	0.16 (0.12 to 0.19)	<0.001	24	0.69 (0.06)	24	0.43 (0.09)	0.27 (0.23 to 0.30)	<0.001	<0.001
	ICP	25	0.72 (0.16)	25	0.55 (0.19)	0.17 (0.11 to 0.22)	<0.001	24	0.66 (0.13)	24	0.31 (0.14)	0.35 (0.28 to 0.42)	<0.001	<0.001
	DCP	25	0.78 (0.14)	25	0.57 (0.17)	0.21 (0.14 to 0.27)	<0.001	24	0.75 (0.10)	24	0.26 (0.14)	0.48 (0.43 to 0.54)	<0.001	<0.001
	Inner Retina	25	0.71 (0.13)	25	0.54 (0.19)	0.17 (0.09 to 0.25)	<0.001	24	0.73 (0.08)	24	0.42 (0.18)	0.31 (0.24 to 0.38)	<0.001	<0.001
Precision	SVC	25	0.71 (0.11)	25	0.76 (0.08)	-0.05 (-0.09 to -0.02)	0.008	24	0.84 (0.08)	24	0.86 (0.07)	-0.02 (-0.04 to 0.00)	0.054	0.08
	ICP	25	0.82 (0.16)	25	0.96 (0.05)	-0.15 (-0.21 to -0.08)	<0.001	24	0.86 (0.09)	23	0.98 (0.03)	-0.12 (-0.15 to -0.08)	<0.001	0.15
	DCP	25	0.84 (0.16)	25	0.95 (0.09)	-0.11 (-0.18 to -0.05)	0.001	24	0.96 (0.05)	24	0.99 (0.01)	-0.03 (-0.06 to -0.01)	0.01	0.010
	Inner Retina	25	0.76 (0.16)	24	1.00 (0.01)	-0.24 (-0.30 to -0.17)	<0.001	24	0.80 (0.07)	24	1.00 (0.00)	-0.20 (-0.23 to -0.17)	<0.001	0.054
Recall	SVC	25	0.84 (0.08)	25	0.54 (0.13)	0.30 (0.25 to 0.34)	<0.001	24	0.81 (0.07)	24	0.46 (0.10)	0.34 (0.30 to 0.39)	<0.001	0.21
	ICP	25	0.87 (0.09)	25	0.57 (0.19)	0.30 (0.24 to 0.36)	<0.001	24	0.76 (0.17)	24	0.31 (0.14)	0.45 (0.37 to 0.53)	<0.001	0.001
	DCP	25	0.93 (0.08)	25	0.59 (0.17)	0.34 (0.28 to 0.40)	<0.001	24	0.78 (0.12)	24	0.26 (0.14)	0.52 (0.45 to 0.58)	<0.001	<0.001
	Inner Retina	25	0.92 (0.07)	25	0.54 (0.19)	0.38 (0.31 to 0.46)	<0.001	24	0.89 (0.06)	24	0.42 (0.18)	0.47 (0.41 to 0.54)	<0.001	0.009
F1-score	SVC	25	0.76 (0.06)	25	0.63 (0.10)	0.14 (0.11 to 0.17)	<0.001	24	0.82 (0.04)	24	0.59 (0.09)	0.22 (0.19 to 0.26)	<0.001	<0.001
	ICP	25	0.83 (0.12)	25	0.69 (0.16)	0.13 (0.08 to 0.19)	<0.001	24	0.79 (0.11)	23	0.48 (0.14)	0.31 (0.25 to 0.38)	<0.001	<0.001
	DCP	25	0.87 (0.10)	25	0.71 (0.14)	0.15 (0.10 to 0.21)	<0.001	24	0.85 (0.07)	24	0.40 (0.17)	0.45 (0.39 to 0.52)	<0.001	<0.001
	Inner Retina	25	0.82 (0.10)	24	0.70 (0.14)	0.12 (0.06 to 0.17)	<0.001	24	0.84 (0.05)	24	0.57 (0.18)	0.27 (0.20 to 0.35)	<0.001	<0.001

Abbreviations: CI, confidence interval; DCP, deep capillary plexus; ICP, intermediate capillary plexus; IOU, intersection-over-union; SD, standard deviation; SVC, superficial vascular complex.

* Total sample size is 50 eyes from 50 individuals. Precision was undefined for the ruled-based method in the ICP and Inner slabs because both the manually graded and rules-based methods did not identify any nonperfusion. Differences, confidence intervals, and P values were calculated from a mixed effects linear model with random participant intercepts. The interaction P value tests the null hypothesis that the difference between the AI and RB methods is the same for scans that were manually graded NPA was less than or equal to versus greater than the median NPA for slab.

Online Table 2. Intraclass Correlation Between Manually Graded and AI or Rules-Based NPA

Anatomic Slab	Method [a] (N=48 eyes, 48 participants)	
	Artificial Intelligence	Rules Based
SVC	0.85 (0.30, 0.95)	0.88 (0.64, 0.95)
ICP	0.86 (0.76, 0.92)	0.67 (0.13, 0.86)
DCP	0.92 (0.86, 0.95)	0.58 (0.21, 0.78)
Inner retina	0.87 (0.15, 0.96)	0.54 (0.02, 0.78)

Abbreviations: ICP: intermediate capillary plexus; DCP: deep capillary plexus; SVC: superficial vascular complex.

[a] Values in parentheses are 95% confidence intervals.

Online Figure 1. Intraclass Correlation of Manually Graded Non-Perfusion Area versus Non-Perfusion Area Graded by Rules-Based or Artificial Intelligence Methods

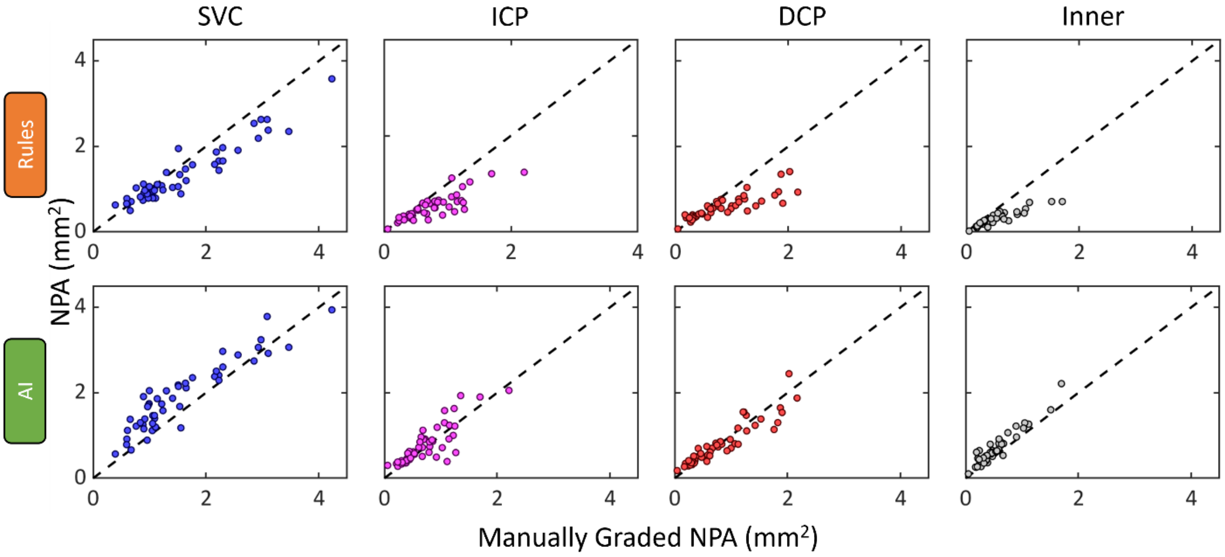

Legend: Scatterplots of manually graded non-perfusion area versus non-perfusion area by grading method (rows) for each slab (columns). Sample size is 48 eyes from 48 participants per slab and method. Abbreviations: AI, artificial intelligence; DCP, deep capillary plexus; ICP, intermediate capillary plexus; RB, rules-based; SVC, superficial vascular complex.

Online Figure 2. Differences in Non-Perfusion Area Graded by Rules-Based or Artificial Intelligence Methods Compared with Manual Grading

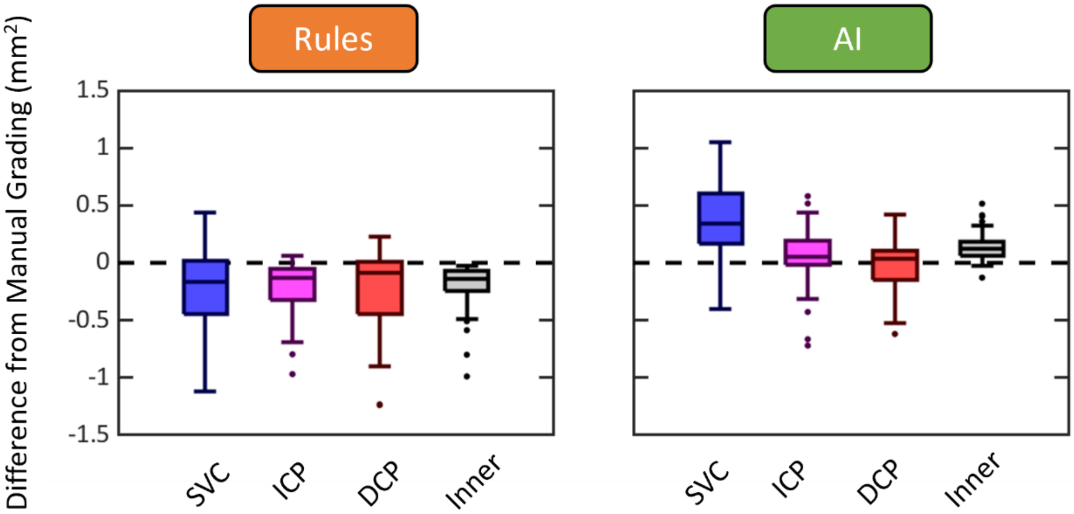

Legend: Boxplot of the difference in non-perfusion area (rules-based = left, artificial intelligence = right) compared with manually graded non-perfusion area. Sample size is 48 eyes from 48 participants per slab and method. Abbreviations: AI, artificial intelligence; DCP, deep capillary plexus; ICP, intermediate capillary plexus; RB, rules-based; SVC, superficial vascular complex.

Online Figure 3. Differences in Non-Perfusion Area Graded by Rules-Based or Artificial Intelligence Methods Versus Manual Grading

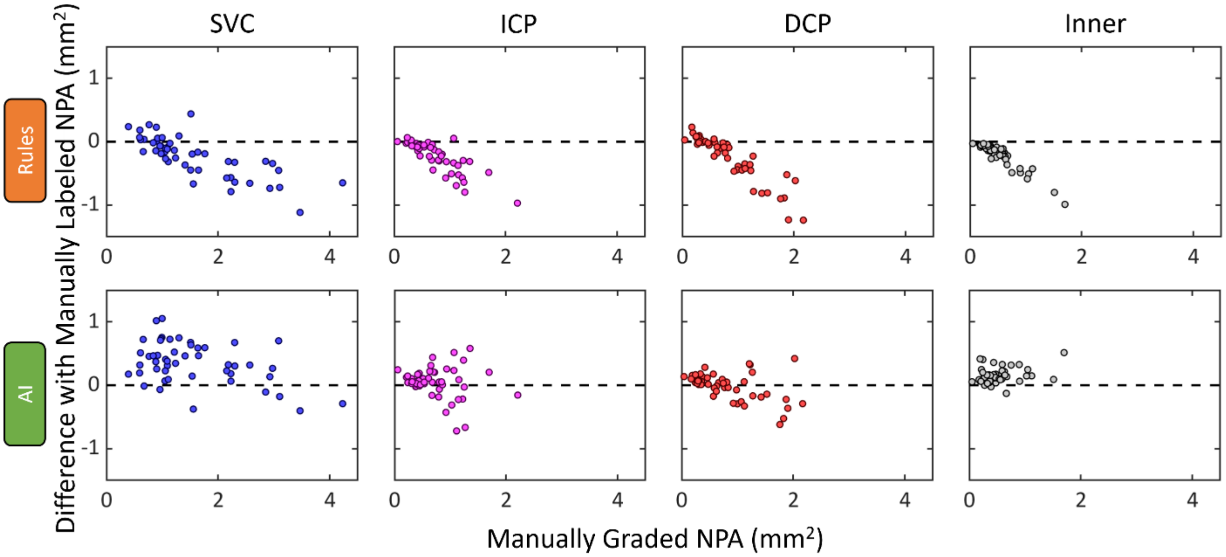

Legend: Scatterplots of manually graded non-perfusion area plotted against the difference (algorithm output) – (manual grading) by grading method (rows) for each slab (columns). Sample size is 48 eyes from 48 participants per slab and method. Abbreviations: AI, artificial intelligence; DCP, deep capillary plexus; ICP, intermediate capillary plexus; RB, rules-based; SVC, superficial vascular complex.

Online Figure 4. Correlation of Diabetic Retinopathy Severity with Non-Perfusion Area by Method and Slab

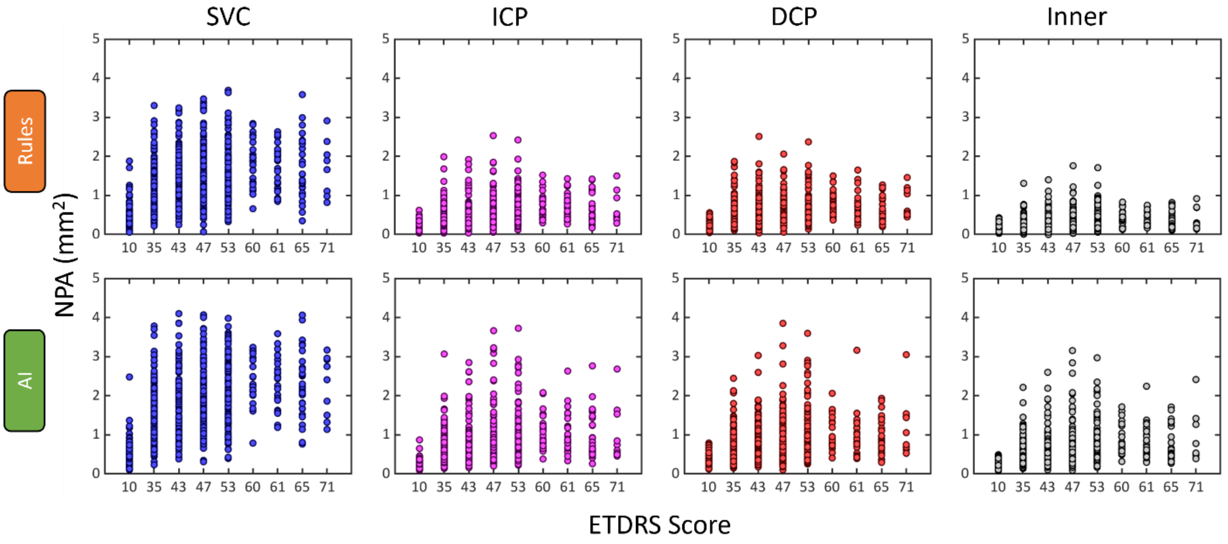

Legend: Scatterplots of diabetic retinopathy severity (ETDRS level) versus non-perfusion area grading by the rules-based (RB) or artificial intelligence (AI) method (rows) by and slab (columns). Sample size is 375 eyes from 375 participants. Abbreviations: DCP, deep capillary plexus; ETDRS, Early Treatment of Diabetic Retinopathy Study; ICP, intermediate capillary plexus; SVC, superficial vascular complex.
